# Advance in remediated of heavy metals by soil microbial fuel cells: Mechanism and application

**DOI:** 10.3389/fmicb.2022.997732

**Published:** 2022-09-29

**Authors:** Yingying Sun, Hui Wang, Xizi Long, Hui Xi, Peng Biao, Wei Yang

**Affiliations:** ^1^Technology Innovation Center for Land Engineering and Human Settlements, Shaanxi Land Engineering Construction Group Co., Ltd. and Xi’an Jiaotong University, Xi’an, China; ^2^State Key Laboratory of Eco-hydraulics in Northwest Arid Region, Xi’an University of Technology, Xi’an, China; ^3^Department of Municipal and Environmental Engineering, School of Water Resources and Hydroelectric Engineering, Xi’an University of Technology, Xi’an, China; ^4^International Center for Materials Nanoarchitectonics (WPI-MANA), National Institute for Materials Science, 1-1 Namiki, Tsukuba, Japan

**Keywords:** soil microbial fuel cell, heavy metal reduction, mass transfer, soil remediation, redox reaction, electric field intensity

## Abstract

In the past decade, studies on the remediation of heavy metals contaminated soil by microbial fuel cells (MFCs) have attracted broad attention because of the self-generated power and their multifield principles such as the extracellular electron transfer (EET) reduction, electromigration for heavy metals removal. However, given the bio electro-motive power from soil MFCs is weak and fluctuated during the remediation, we need to comprehensively understand the origination of driving force in MFC based on the analysis of the fundamental rationale of ion moving in cells and improve the performance *via* the appropriate configurations and operations. In this review, we first described the structures of soil MFCs for heavy metals remediation and compared the advantages of different types of configurations. Then, based on the theoretical models of heavy metal migration, enrichment, and reduction in soil MFCs, the optimization of soil MFCs including the length of the remediation area, soil conductivity, control of electrode reaction, and modification of electrodes were proposed. Accordingly, this review contributes to the application of bioelectrochemistry to efficiently remove heavy metals from soils.

## Introduction

According to the Ministry of Natural Resources of China, there were 53,598 mines by the end of 2020 (Ministry of Natural Resources, PRC, 2020b), while abandoned open-pit mines in key watersheds and regions, such as the Yellow River Basin and the Fenwei Plain, have significantly contributed to heavy metal pollution (Ministry of Natural Resources, PRC, 2020a). Metal (loid) exploitation and mining activities have led to increased toxicity of ecosystems and threaten human health when their residuals were released into the water, soil, air, or food chain (Liu et al., 2019). Over the last three decades, various *in-situ* and *ex-situ* soil remediation techniques have been developed to remediate heavy metal-contaminated soils, which can be grouped into soil washing, soil replacement, electrokinetic remediation, chemical fixation, chemical leaching, phytoremediation, and bioremediation (Liu et al., 2017; Ali et al., 2022). However, these physicochemical techniques are energy-intensive, cost-effective, and emit greenhouse gases into the atmosphere. Although the biological treatment techniques are economical and environmentally friend, they are greatly limited in the treatment of heavy metals with low bioavailability (Rajendran et al., 2022).

Based on the extracellular electron transfer (EET) process discovered in electroactive bacteria (EAB), such as the *Geobacter* sp., soil microbial fuel cells (MFCs) are increasingly developed and studied in recent years and have attracted significant attention as an environmentally sustainable bioelectrochemical technology (Li et al., 2017; Chen et al., 2021). Soil MFCs offer an alternative approach by providing electron donors/acceptors, thereby enhancing bioremediation processes, and migrating while reducing heavy metals in soil (Wang et al., 2020a; Zhang et al., 2020b). The principle of remediating heavy-metal-contaminated soils *via* MFCs lies in the fact that the microorganisms in the deeper subsoil (served as an anode in an anaerobic environment) can oxidize organic matter to generate electrons. It is followed by the electrons transfer to reach the aerobic surface layer of the soil (cathode) *via* an external circuit to the electron acceptor (oxygen or heavy metal). The redox reaction couples organic matter oxidation–oxygen/heavy metal reduction and accompanies the current generation. Simultaneously, heavy metals migrate from the subsoil to the surface layer under the electric field generated from the soil MFC (Wang H. et al., 2016). The remediation of heavy metals in soil by MFC mainly accomplished in two ways: (1) reducing the bioavailability of heavy metals by electrical migration from soil (Wang H. et al., 2016; Zhang et al., 2020a; Hemdan et al., 2022). (2) bioelectrochemical reduction of heavy metal to the low valence, associated with the precipitation/detoxication in the MFCs (Habibul et al., 2016b; Kabutey et al., 2019).

Nonetheless, heavy metals removal *via* soil MFCs is in its infancy and there are some issues to be studied urgently: (1) the effect of soil’s physical and chemical properties on MFC’s electricity generation and heavy metal removal; (2) the relationship between electricity generation and heavy metal migration, enrichment, and reduction; (3) optimizing the construction and components of soil MFCs. Hence, in the present review, the mechanisms of soil MFCs involved in the current generation and heavy metal remediation are discussed. In addition, theoretical models of heavy metal migration, enrichment, and reduction in soil MFCs are analyzed. It is highly expected that this review can provide useful information and suggestions to promote the practical and sustainable application of this technology.

## The soil MFCs with different structural configurations and soils

**Figure 1:**
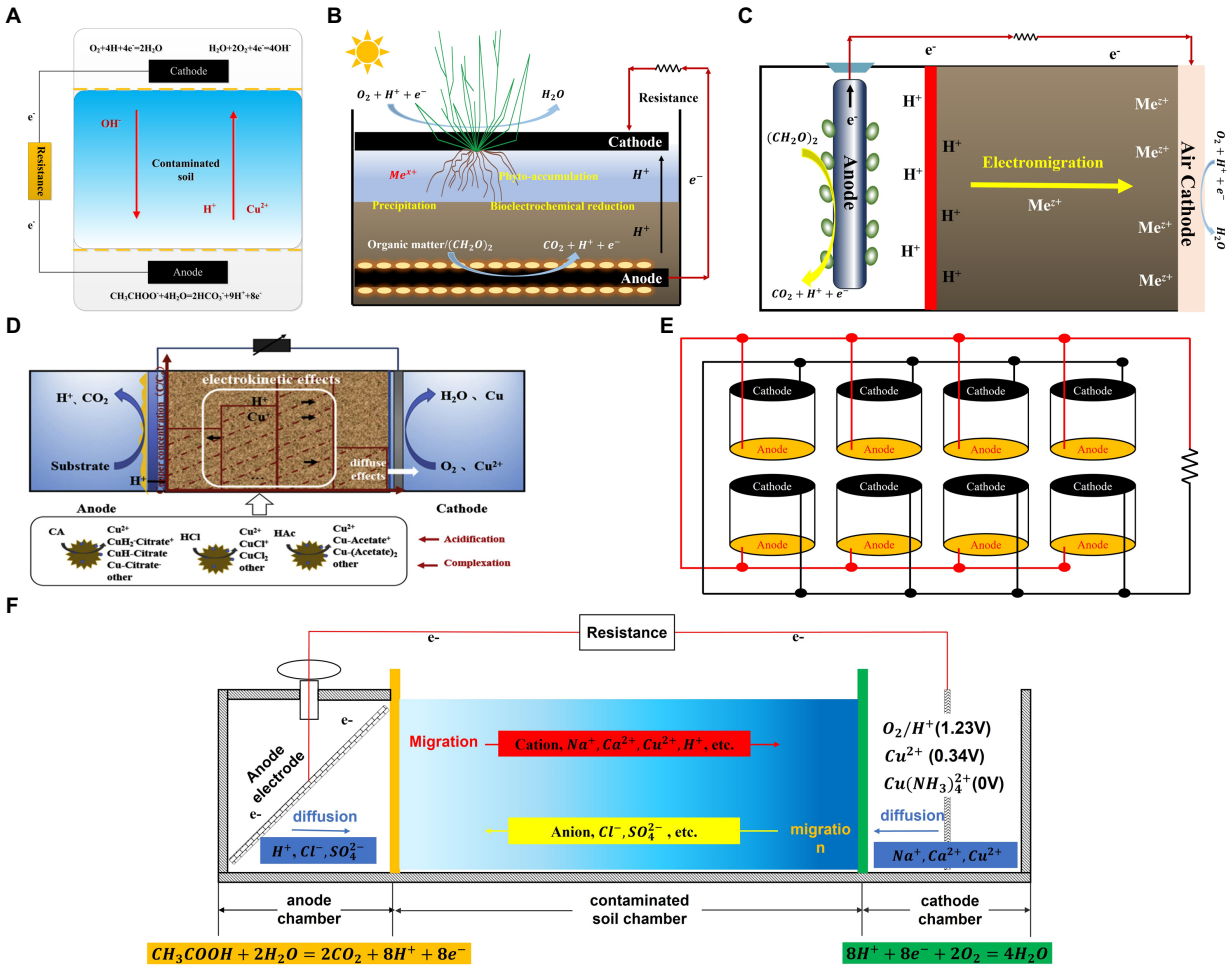
Typical configurations of soil MFCs. **(A)** single-chamber (Wang H. et al., 2016), **(B)** plant (Guan et al., 2019b) **(C)** two-chamber (Habibul et al., 2016a), **(D)** three-chamber (Zhang et al., 2020a), **(E)** stack MFCs (Dziegielowski et al., 2021), and **(F)** Mass transfer of multi-ions in soil MFC induced by diffusion and migration. All panels are with the permission from publishers’ copyright.

The MFC established in the soil is developed based on the rationale of the fuel cell. The anode harvests electrons from the degraded organics, such as acetate, and amino acids in the soil by EAB. Then, the cathode receives electrons through an external circuit where oxygen is reduced to water, associated with the migration of protons (Dunaj et al., 2012). As shown in Figure 1, various types of soil MFCs have been designed for heavy metal remediation. The single-chamber soil MFCs take the advantage of redox potential/oxygen gradients between the subsoil and the topsoil to set the anode and cathode, respectively, while the proton exchange membrane can be saved (Huang et al., 2020). Wang H. et al. (2016) established a single-chamber soil MFC to study the migration of copper. The six sections along the soil revealed an obvious accumulation of Cu^2+^ near the cathode from 150 to 250 mg/kg, indicating the feasibility of the single chamber soil MFC (Figure 1A). However, heavy metal migration is not persistent because of the deficiency of carbon sources which leads to the power density weakening. Meanwhile, to replenish the carbon source, exterior organics such as straw, and sodium acetate were added to soil MFC. In addition, heavy metals cannot migrate from the soil to the cathode (such as activated carbon) because of heterogeneity, thereby minimizing the remediation effects (Wang H. et al., 2016). Hence, to enhance heavy metal removal, plant-microbial fuel cells (PMFCs), where plants have been placed in the topsoil and the root exudates supplied organics to EAB in the anode (Figure 1B), have been developed and used for heavy metal contaminated waters and soils (Guan et al., 2019b; Li et al., 2020). Moreover, despite the cathodic bioelectrochemical reduction, the direct reduction by reducing microorganisms, plant uptake, and adsorption by electrodes, enriched the pathway and efficiency for heavy metal removal (Habibul et al., 2016b).

Given that the EAB in single-chamber soil MFC would be inactive because of the toxicity of heavy metals and also would be difficult to be accumulated in soil located anode, the double-chamber air-cathode soil MFCs were constructed and were able to generate a stronger electrical field capable of powering electrokinetic remediation (Figure 1C; Habibul et al., 2016a; Wang C. et al., 2016). Compared to the single-chamber soil MFC, the anode and cathode chambers here are separated by a proton/cation exchange membrane. The resultant removal efficiencies were 31.0% for Cd, 41.1% for Pb, and 99.1% for Cr, respectively (Habibul et al., 2016a; Wang C. et al., 2016). However, the heavy metals enriched near the cathode regions could not be processed further owing to the heterogeneity of soil and electrode, precipitation, metal species characteristics, etc. Thereby, to further enhance the cathode reduction, the three-chamber soil MFCs consisting of an anode, a contaminated soil chamber, and a cathode chamber for heavy metal removal were established (Figure 1D; Wang et al., 2020a; Zhang et al., 2021b). This type of soil MFCs not only supports heavy metal migration from the soil toward the cathode but can also reduce the heavy metals in the cathode by adjusting the current or voltage generated. Regarding the use of soil MFCs as a power source and their efficient and stable performance over long periods, Dziegielowski et al. (2020, 2021) have developed and scaled a stack of soil MFCs to generate sufficient renewable energy for powering a water treatment electrochemical reactor, as a way of using soil MFCs as a power source (Figure 1E).

Soil is a heterogeneous multiphase system, and its types affect the performance of soil MFCs. It was reported that the red soil generated a higher current than the fluvo-aquic soil while showing a higher Cr(VI) removal efficiency and cathode efficiency since there were more electron acceptors in red clay than in fluvo-aquic soil, such as Fe(III) (Wang C. et al., 2016). These electron acceptors would compete with the reduction of Cr(VI) while hindering the removal of Cr(VI) by MFC. However, more electrons will enhance the electricity-generating performance of MFC. And red soil contains more clay particles, which have stronger adsorption to Cr(VI), thus hindering the migration of Cr(VI) in the soil to the cathode (Wang C. et al., 2016). Moreover, Zhang et al. (2020b) believed that the physical and chemical properties of different soils would affect the removal of heavy metals in the soil MFCs. Decreasing soil pH, total organic carbon, and cation exchange capacity could promote the heavy metal of diffusion and electromigration. Higher soil electrical conductivity and pH could improve the electricity generation performance, which enhanced the electromigration of copper ions (Habibul et al., 2016a; Zhang et al., 2020b).

Ion migration is greatly inhibited when the soil moisture content is unsaturated; thus, regardless of the soil MFC type, considerable limitations exist when remediating contaminated soil (Popat and Torres, 2016; Kumar et al., 2017). As the anode and cathode pH significantly differ, protons generated in the former are transported more slowly to the latter when compared to the inverse route (Gil et al., 2003). This is because the cathode readily accepts electrons and is reduced to hydroxyl ions (De Schamphelaire et al., 2008). Consequently, heavy metals are precipitated from hydroxides on/near the cathode, restricting the migration of metal ions (Hicks and Tondorf, 1994). Thus, the construction of *in-situ* soil MFCs in contaminated soils remains a challenge. The low performance of soil MFCs for the removal of heavy metals is shown in Table 1. It is difficult to maintain sufficient soil organic matter content for sustaining the metabolism of electroactive microorganisms in a long-term operation. Whereas laboratory studies often add simple carbon sources to the soil for electricity production, and heavy metal removal and migration (Huang et al., 2020; Zhang et al., 2020b, 2021a); the addition of such carbon sources is not sustainable for *in situ* soil pollution remediation and engineering applications. Therefore, the configuration of soil MFC should be further studied along with the in-depth study of the EET mechanisms and its coupled redox reactions of heavy metals.

**Table 1:** Soil MFCs for the removal of heavy metals.

Heavy metals	Configuration	MFC electrode	Power density	Carbon source/electron donor	Removal/reducing efficiency and time	Driving force	References
1	Single-chamber	Granular activated carbon for anode and cathode	65.77 mW/m^2^	Sodium acetate	Maximum 20%, 56 days	Electric migration	Wang H. et al., 2016
Cr		Carbon felts or graphite carbon felts for the anode and the cathode	469.21 mV	Root exudates	99%, 53 days	Electric migration, adsorption, and reduction	Guan et al., 2019b
Cr		Carbon felts or graphite carbon felts for the anode and the cathode	N/A	Root exudates	2.34-fold accumulated in cathode; 1.89-fold accumulated in plant root (near cathode) after 10 months	Electric migration, adsorption, and reduction	Guan et al., 2019a
Cd		Carbon felts for the anode and the cathode	22.93 mW/m^2^	Sodium acetate	Maximum 30%, 50 days	Electric migration	Huang et al., 2020
Zn, Pb		Graphite felt pads for the anode and the cathode	25.7 mW/m^2^	Straws	Maximum 30% (Pb) 15% (Zn), 50 days	Electric migration	Song et al., 2018
Cd, Cu, Cr, and Ni		Three carbon felt pads for the anode and the cathode	22.2 ± 1.6 mW/m^2^	Root exudates	35.1%, 32.8%, 56.9%, and 21.3% (Cd, Cu, Cr, and Ni in rice grains), 110 days	Electric migration	Gustave et al., 2020
As		Three carbon felt pads for the anode and the cathode	123.0 ± 2.2 mW/m^2^	Organics in paddy soil	37.5% in pore water, 60 days	Electric migration	Gustave et al., 2019
As		Three carbon felt pads for the anode and the cathode	12.0 mW/m^2^	Organics in paddy soil	47% at the anode, 50 days	Electric migration	Gustave et al., 2018
Cd, Cr	Double-chamber	Carbon brushes for the anode and carbon cloth for the cathode	48.8 mW/m^2^	Sodium acetate	Maximum 7.6% (Cr)12.1% (Cd), 50 days	Electric migration	Wang et al., 2020b
Pb, Cd		Graphite granules for the anode and carbon felt for the cathode	7.5 mW/m^2^	Sodium acetate	Maximum 44% (Pb), 108 days; 31% (Cd), 143 days	Electric migration	Habibul et al., 2016a
Cr		Porous carbon felts for the anode and the cathode	200–300 mW/m^2^	Sodium acetate	Maximum 35% (Cr), 16 days	Reduction, adsorption	Wang C. et al., 2016
Zn, Cd	Three-chamber	Graphite for anode and Graphite mesh/Pt coated for cathode	0.4 mA/cm^2^	Sodium acetate	25% (Zn), 18% (Cd), 78 days	Electric migration	Chen et al., 2015
Cu		Carbon felt for anode and stainless-steel mesh for cathode	222.72 mW/m^2^	Sodium acetate	2.33-fold accumulated in soil, 100% removal in the cathode, 56 days	Electric migration, reduction	Wang et al., 2020a
Cu		Carbon felt for anode and stainless-steel plate for cathode	58.34 mW/m^2^	Sodium acetate	41%, 74 days	Electric migration	Zhang et al., 2020a
Cu		Carbon felt for anode and stainless-steel plate for cathode	65.80 ± 1.29 mW/m^2^	Sodium acetate	1.5-fold accumulated in soil, 100% removal in the cathode, 21 days	Electric migration, reduction	Zhang et al., 2021b
Cu		Carbon felt for dual anode and stainless-steel plate for cathode	42.48 mW/m^2^	Sodium acetate	24.1%, 56 days	Electric migration,	Zhang et al., 2021a
Cu		Carbon felt for anode and stainless-steel plate for cathode	54 mW/m^2^	Sodium acetate	19.3% ± 0.8%, 63 days	Electric migration,	Zhang et al., 2020b

## The model of migration, enrichment, and reduction process of heavy metals in soil MFCs

The underlying mechanisms of soil MFCs for heavy metal removal are based on electromigration and electroosmotic flow (Chen et al., 2015; Habibul et al., 2016a). It has been reported that Cu, Cd, and Pb gradually accumulate along the soil from the anode to the cathode owing to the established electric field (Figure 1F; Habibul et al., 2016a; Wang H. et al., 2016). However, even though the migration and movement of heavy metals have been observed in previous studies, experimental trials targeting effective improvement are still lacking, in part related to the ambiguity of rationale for the underlying process driving MFC cells. Conventionally, the process of electrokinetic remediation (EKR) of soil is regarded as an analogy to soil MFC. However, the differences underlying the externally supplied power of EKR and the self-constructed electric field inside the soil MFC cell are commonly neglected, where: (1) the EKR process was conducted under an electric field strength much higher than that of the soil MFC (Liu and Logan, 2004; Li et al., 2014); or (2) the distinct mechanisms are largely ignored as the redox reactions in EKR are the electrolysis of water, while the reactions in soil MFCs are established by the potential difference between the oxidation of organics and the reduction of oxygen/heavy metals (Logan et al., 2006).

The overall mass transfer of heavy-metal ions is driven by the electrochemical potential of the electric field. According to the bias of the potential and additional velocity of the solution, the flux of the ions is described by the Nernst–Planck equation (Bard and Faulkner, 2001):


(1) $$J_{i}=-\frac{Z_{i}F}{RT}D_{i}C_{i}\nabla \Phi +C_{i}V-D_{i}\nabla C_{i}$$

where *Z_i_* represents the valence of ions, *F* is the Faradaic constant (C/mol), *R* is the gas constant (J/K·mol), *T* is the temperature (K), *D_i_* is the diffusivity of the ions (m^2^/s), *C_i_* is the concentration (mol), *Φ* is the electric field strength (V/m), and *V* is the fluid velocity (m^3^/s). In general, heavy metals can migrate from the anode to the cathode *via* the electrical field capable of powering electrokinetic remediation. In addition, despite the cathodic bioelectrochemical reduction, the direct reduction by reducing microorganisms, plant uptake, and adsorption by electrodes, enriched the pathway and efficiency for heavy metal removal.

## Optimizing soil MFC operation and configuration for heavy metal removal

For the convenience of the ensuing presentation, copper is taken as an example of the target pollutant (Figure 1F). Electromigration is determined by the valence of ions as well as the external electric field and is regarded as the main driving force in EKR for heavy metal movement (Acar et al., 1995; Han et al., 2021). For soil MFCs, anode potential is primarily controlled by electroactive bacteria and the reduced species on the electrode. Accordingly, the anode potential can be estimated as *E_anode_* = −0.32 V (for NAD^+^/NADH redox pair), while the cathode potential as *E_cathode_* = 0.4 V (for O_2_ 4 electrons reduction; Logan et al., 2006). Importantly, this voltage of ~0.5 V is ≥200 times less than that used for the EKR process. Furthermore, because of the large internal resistance contributed by the long distance of the remediation area, the polarization further reduces the voltage (Wang H. et al., 2016). Nevertheless, Ca^2+^, Na^+^, and K^+^ ions in the soil electrolyte/buffer compete as electron carriers, thereby decreasing the transference number of heavy metals and undermining the electric migration capacity of heavy metals (Bard and Faulkner, 2001). As such, migration in the soil is severely impaired in MFCs.

As the electromigration and electroosmotic flow caused by strong voltage are predominant in the EKR process, corresponding discussions on the diffusion process are usually omitted. However, the importance of diffusion as the driving force for heavy metal movement must be emphasized in soil MFCs. Diffusion is caused by a concentration gradient, originating from the consumption of redox species on the electrode. In a soil MFC cathode, the competition between oxygen and Cu^2+^ reduction simultaneously dictates the priority of diffusion and migration. The reduction O_2_ potential is higher than that of Cu^2+^, preferentially favoring the O_2_ reduction. Similarly, a comparatively large amount of O_2_ over Cu^2+^ supports a slightly higher current reduction (i.e., the current output) for migration. In contrast, the weak Cu^2+^ reduction rate retards the formation of a concentration gradient for heavy metals, thereby impairing the diffusion process. Consequently, the control of soil MFCs for heavy metal removal requires further consideration, and such perspectives for system optimization are given below:

(1) The construction of the soil MFC should be designed depending on the length of the remediation area. Despite the intrinsic character of the redox reaction on the electrode, the electrical strength is directly related to the length of the cell. Zhang et al. (2021b) discovered that the internal resistance of soil decreased from 1,176 Ω (20 cm) to 583 Ω (5 cm; corresponding to 2.68 mV·cm^−1^ and 8.92 mV·cm^−1^, respectively), while shorter lengths of soil MFC were associated with stronger total copper migration removal rates. After 63 days of remediation, the removal rates of acid-extractable copper in the soil were 42.50% and 12.40%, respectively. Considering that a higher internal resistance significantly affected cell polarization and deteriorated voltage output, the length of the soil MFC should be controlled at approximately 5 cm (Zhang et al., 2021b).
(2) Soil conductivity should be controlled within an appropriate range to enhance MFC voltage output while ensuring the electromigration efficiency of heavy metals. Zhang et al. (2020a) compared the influence of the physical and chemical properties of soil on MFC power generation, observing that a higher soil conductivity promoted the current and decreased electrode polarization. However, the Phosphate-Buffered saline or electrolyte used in ordinary liquid MFCs is not suitable for soil addition. In fact, the widespread and abundant non-reaction ions in the MFC potential range (e.g., Ca^2+^, Na^+^, and K^+^) function as supporting electrolytes in soil MFCs, severely minimizing the percentage of ion current from heavy metals and suppressing electromigration (Chen et al., 2021). To balance the conductivity of soil and the migration efficiency, desorption agents, such as small molecular organic acids (e.g., citric, tartaric, or acetic acid), inorganic acids (e.g., hydrochloric or nitric acid), and synthetic chelating agents (e.g., ethylenediaminetetraacetic acid), can effectively dissolve heavy metals in the acid extractable state, thereby increasing the conductivity of soil MFCs, and resulting in the higher power generation while simultaneously maintaining an increased migration rate of heavy metals (Zhang et al., 2020a).
(3) The electrode reaction rate should be controlled to overcome the competition between different electron acceptors. Clear competition between O_2_ and Cu^2+^ occurs because the standard reduction potential (vs. SHE) of O_2_/H_2_O (1.229 V) is much higher than that of Cu^2+^/Cu (0.337 V) or Cu with the anionic ligand Cu(NH_3_)_4_^2+^/Cu (0.0 V; Figure 1F; Bard and Faulkner, 2001). Thermodynamically, O_2_ (1.229 V) was reduced on the electrode prior to Cu^2+^. When the reaction rate (current) of the soil MFC cathode is slow, the electrons transferred to the cathode preferentially react only with the relatively abundant dissolved oxygen (not with Cu^2+^). Conversely, when the reaction rate of the cathode is relatively fast, the cathode is controlled by the electrode and becomes diffusion-controlled. In this case, the concentration of oxygen and protons on the electrode surface is low and is difficult to replenish. The competition with Cu^2+^ is in turn mitigated, and significantly more Cu^2+^ is reduced to be associated with the higher current. Generally, to achieve a high removal efficiency of heavy metals in soil MFCs, the reaction rate of the electrode should be initially controlled at a low level (for example, loading a large external resistance) to rapidly establish a higher electric field strength for Cu^2+^ mitigation to the cathode. Then, a fast electrode reaction rate was applied to effectively reduce the heavy metals on the cathode, thereby accelerating Cu^2+^ reduction. In addition, multi-heavy metal ions still would be migrated or reduced since the electric field generated by soil MFC had no selectivity for the driving of charged ions (Zhang et al., 2022). Wang et al. (2020b) found that the interaction between negatively charged chromium and positively charged lead in the soil had no major effect on hindering migration. Moreover, the remediation performance of composite heavy metal contaminated soil was better than that of single heavy metal contaminated soil. It should be noted that some heavy metals with negative potential (e.g., Pb^2+^, Cd^2+^) could not be reduced unless performed in stacked microbial electrolysis cells with high series voltage (Zhang et al., 2015; Wang Q. et al., 2016).
(4) Designing the electrode in soil MFC to boost the energy conversion for heavy metal removal in soil MFC. Carbon materials are commonly selected to be the anode owing to their biocompatibility and chemical stability. For instance, after high-temperature pyrolysis, the biochar material develops cracks to form a pore structure, which greatly increases the specific surface area (Huggins et al., 2014). In addition, biochar owed good electrical conductivity and capacitance to accommodate electrons, which has greatly promoted the interspecific electron transfer (Sakhiya et al., 2020). Moreover, it was pseudo-discovered that doping metal oxides and conductive polymers with biochar can greatly improve the capacitance characteristics and lead to an increase in the current output of MFC (Thines et al., 2017; Norouzi et al., 2020). This pseudo-capacitance increases the specific capacitance value of the electrode by a factor of 10–100 times compared to the ordinary electric double-layer capacitance, greatly improving the electron storage capacity of the interface (Liang et al., 2021). Meanwhile, these material modifications also introduce a large number of electrons transfer active sites (Feng et al., 2014). The high electrical conductivity, fast reversible redox ability of metal oxides, and abundant functional groups (such as amino and catechol functional groups) on the surface of conductive polymers 4–6 are favorable for the formation of active sites for electron transfer (Du et al., 2017).

Overall, three factors were observed when using soil MFCs to remediate heavy metal-contaminated soil. Firstly, improving soil conductivity and increasing the output voltage/current of soil MFCs will promote the migration of metal ions in the soil and the efficiency of cathode reduction; Secondly, more electrons from the anode can be used to reduce heavy metals by adjusting the cathode oxidation–reduction potential, thereby facilitating the reduction or morphological changes of heavy metals, reducing the concentration of heavy metals in the cathode, and increasing the transfer of heavy metals from the anode to cathode; and finally, the range of available remediation areas should be controlled because the electric field intensity generated by soil MFCs is much smaller than that generated by electric remediation.

## Conclusion

While the bio-electrochemical method derived from soil MFCs has been developed in the last decades, the soil MFCs suffered from the constrained current, corresponding to the potential for heavy metal removal/reduction. Here, starting from the discussion of the configuration of MFCs, we collectively concluded the power generation and their removal of heavy metals in single-, double-, and triple-chamber soil MFCs. Meanwhile, by comparing the process of electrokinetic remediation (EKR) of soil, the migration, enrichment, and reduction process in soil MFCs were evaluated. Then we proposed the method to optimize soil MFCs operation/construction for heavy metal removal. Generally, our review concludes the perspective and challenge of the soil MFCs and would guide the improvement of soil MFC for heavy metal removal.

## Author contributions

HW and XL contributed to the conception and design of the study. YS and PB wrote sections of the manuscript. HX and WY drew these pictures and checked the language. All authors contributed to manuscript revision, read, and approved the submitted version.

## Funding

This work was financially supported by Technology Innovation Center for Land Engineering and Human Settlements, Shaanxi Land Engineering Construction Group Co., Ltd. and Xi’an Jiaotong University (2021WHZ0094), National Natural Science Foundation of China (42107030), Natural Science Basic Research Program of Shaanxi (2020JQ-617), and the postdoctoral program from Japan Society for the Promotion of Science (P20105).

## Conflict of interest

YS, XH, BP, and YW were employed by the company Shaanxi Land Engineering Construction Group Co., Ltd.

The remaining authors declare that the research was conducted in the absence of any commercial or financial relationships that could be construed as a potential conflict of interest.

The authors declare that this study received funding from Shaanxi Land Engineering Construction Group Co., Ltd. The funder was not involved in the study design, collection, analysis, interpretation of data, the writing of this article or the decision to submit it for publication.

## Publisher’s note

All claims expressed in this article are solely those of the authors and do not necessarily represent those of their affiliated organizations, or those of the publisher, the editors and the reviewers. Any product that may be evaluated in this article, or claim that may be made by its manufacturer, is not guaranteed or endorsed by the publisher.
